# Monitoring of Polychlorinated Biphenyls (Pcbs) Contamination in Milk and Dairy Products and Beverages in Türkiye: A Public Health Perspective

**DOI:** 10.3390/foods14203544

**Published:** 2025-10-17

**Authors:** Oltan Canlı, Barış Güzel, Merve Türk, Burhan Basaran

**Affiliations:** 1 Climate Studies and Water Management Research Group, Climate and Life Vice Presidency, TUBITAK Marmara Research Center, 41470 Gebze, Kocaeli, Türkiye; oltan.canli@tubitak.gov.tr (O.C.); guzelbaris08@gmail.com (B.G.); turkmerve89@gmail.com (M.T.); 2 Department of Environmental Engineering, Kocaeli University, Umuttepe Campus, 41275 Izmit, Kocaeli, Türkiye; 3 Department of Nutrition and Dietetics, Faculty of Health Sciences, Recep Tayyip Erdogan University, 53100 Rize, Rize, Türkiye

**Keywords:** PCBs contamination, packaging safety, dietary exposure, risk assessment

## Abstract

In this study, the presence of seven polychlorinated biphenyl (PCB) congeners proposed by ICES-7 (International Council for the Exploration of the Sea) (PCBs 28, 52, 101, 118, 138, 153, and 180) in milk, dairy products, and beverages was investigated, and potential risks to consumer health were assessed. A total of 130 samples were analyzed using gas chromatography–mass spectrometry (GC–MS/MS). Most PCBs levels were below the limits of detection and quantification, but trace amounts, particularly of PCB 153 and PCB 180, were detected. Overall, 35% of milk and dairy products and 20% of beverage samples exceeded the reference limits for ICES-7, with higher accumulation observed in high-fat dairy products. Packaging type also appeared to influence contamination levels. The study findings indicate that PCBs contamination levels may vary depending on product type, content, production method, and packaging structure. Three consumption scenarios were modeled for children and adults, and the estimated daily intake (EDI) was calculated. All hazard index (HI) values found to be below 1. This result suggests no significant non-carcinogenic health concern across the examined products and packaging types. Nevertheless, given the persistence and bioaccumulation potential of PCBs, continuous monitoring remains essential.

## 1. Introduction

Milk and dairy products are not only a primary source of essential nutrients but also a food group rich in biologically active components that directly affect metabolic functions [1,2]. Thanks to their high bioavailability of calcium and high-quality protein, they play important roles, particularly in maintaining growth, bone mineralization, and muscle function [3]. Furthermore, the probiotic microorganisms found in fermented dairy products contribute to strengthening the gut microbiota and immune response [4]. Today, milk and dairy products are increasingly considered functional foods in modern nutritional approaches due to their properties that support macronutrient balance and help prevent chronic diseases [5,6]. Milk adulteration and contamination constitute a serious global concern, since dairy products are consumed daily by billions of people. Adulterants such as melamine or starch, and contaminants including antibiotics, pesticides, heavy metals, and mycotoxins, present significant health risks [7,8]. Recent advances in analytical methods, such as chromatographic techniques coupled with mass spectrometry (LC–MS/MS, GC–MS/MS), spectroscopic approaches (FTIR, NIR, Raman), point-of-use sensor and biosensor-based platforms, have greatly improved the detection of adulterants and contaminants [9,10,11]. Moreover, both the integration of chemometrics and artificial intelligence and a configuration combining a laser diode with a PC-interfaced digital CMOS camera have enhanced the speed and reliability of milk safety assessments [12,13].

Packaged beverages such as fruit juice, cola, and soda have become an important component of the daily diet, especially with the rise in urbanization, fast-paced lifestyles, and externally dependent eating habits [14,15]. Water is critical for healthy hydration [16]. While fruit juices are perceived as a natural and nutritious option by most consumers, they can be controversial due to their added sugars [17]. On the other hand, sugary, carbonated beverages such as soft drinks are frequently consumed due to their taste, addictive effect, and sociocultural factors. However, their high content of sugar, artificial additives, and excess calories have been linked to diabetes and cardiovascular disease [18,19].

Polychlorinated biphenyls (PCBs) are persistent organic pollutants originating from industrial activities, characterized by high resistance to environmental degradation and by their tendency to bioaccumulate and biomagnify within the food chain, posing significant risks to ecosystems and human health [20,21]. The main sources of PCBs include industrial emissions, waste incineration plants, and fossil fuel combustion, and after being released into the atmosphere, they are transported into the food chain through air, water, and soil [22,23]. Consumption of contaminated vegetables or food products obtained from animals fed with these vegetables is a significant source of PCBs exposure [24,25]. Due to their highly lipophilic properties, PCBs accumulate in fat-rich foods, particularly milk, meat, eggs, fish, and vegetable/animal oils, and are directly absorbed into the human body through consumption of these foods [26,27]. Furthermore, packaging materials containing PCBs can constitute a secondary source of contamination through contact with food [28].

Studying PCBs in food is critical for both public health and environmental safety. Extensive scientific research has shown that long-term exposure to PCBs is associated with liver toxicity, immune system suppression, thyroid hormone disorders, reproductive disorders, neurodevelopmental disorders, and some types of cancer [29,30]. In particular, PCBs can pass to the fetus via the placenta during pregnancy or be transferred to babies through breast milk during breastfeeding [31]. This poses a significant public health problem that can cause serious developmental problems in vulnerable groups, especially infants and young children [32]. In addition, certain PCB congeners are classified as Group 1 carcinogens by the International Agency for Research on Cancer (IARC) [33]. Considering these risks, regular monitoring and analysis of PCB residues in food are important for detecting violations of reference limits, ensuring food safety, protecting consumer health, and supporting risk assessment models. Monitoring studies also play a vital role in revealing the effects of environmental pollution (industrial zones, waste disposal sites, feed sources) on the food chain and in developing sustainable production policies.

The aim of this study is to determine PCB levels in milk, dairy products, and beverages sold in Türkiye, and to assess exposure resulting from their consumption in terms of non-carcinogenic health risks. This study fills a significant gap in the literature by providing systematic and up-to-date data on PCBs contamination in dairy products and beverages. The findings will contribute to the development of national food safety policies and improved regulations. Finally, the study has the potential to become a pioneering reference in the field with both its methodological approach and comprehensive dataset, informing new strategies to reduce foodborne PCBs exposure.

## 2. Methodology

### 2.1. Materials

In this study, there are a total of 130 products with high consumption levels, characterized by well-known brands. The samples were purchased from large supermarket chains and local markets in different regions to reflect the diversity of products commonly available to consumers. They covered multiple brands and product categories, ensuring a representative coverage of the most widely consumed milk and dairy products and beverages, although not exhaustive of all brands nationwide (Türkiye). A total of 51 milk and dairy product samples were sampled: UHT milk (n = 12), children’s milk (n = 17), yogurt (n = 12), kefir (n = 6), and ayran (traditional product) (n = 4). A total of 51 beverages samples were sampled: soft drinks (n = 24), fruit juice (n = 15), energy drinks (n = 9), water (n = 8), cold coffee (n = 8), ice tea (n = 7), lemonade (n = 4), and traditional turnip juice (n = 4). Among dairy product samples, samples 1–22 were sold in plastic packages (polyethylene and polystyrene), while samples 23–51 were sold in Tetra Pak packages. Among beverage samples, samples 52–62, 76, 77, 91, 100–107, and 123–130 were sold in plastic packages (polyethylene, polypropylene, and polyethylene terephthalate); samples 63–75, 92–99, and 108–122 in tin packages; and samples 78–90 in Tetra Pak packages. More details about the products are shown in Tables S1 and S2.

### 2.2. Analysis

#### 2.2.1. Reagents and Standards

In this study, a standard solution of PCB-Mix 3 (100 ng/µL in iso-octane), comprising seven indicator PCB congeners (PCB 28, PCB 52, PCB 101, PCB 118, PCB 138, PCB 153, and PCB 180), was obtained from Dr. Ehrenstorfer GmbH (Augsburg, Germany) in a 10 mL amber glass vial. Internal standards (ISs) containing PCB 29 and PCB 198 (100 ng/µL in cyclohexane) were also supplied by Dr. Ehrenstorfer GmbH. All solvents used—acetone and n-hexane (purity 99.0–99.8%)—were of analytical grade and purchased from Merck (Darmstadt, Germany), while tetradecane (≥99% purity) was supplied by Sigma-Aldrich (London, UK). Post-extraction sample clean-up was carried out using SampliQ Silica (500 mg, 3 mL, 50/pk) and Silica SCX-Box (500 mg, 3 mL, 50/pk) solid-phase extraction columns (Agilent Technologies, Avondale, AZ, USA). High-purity helium gas (99.9%) was employed as the carrier gas in all GC-MS/MS analyses.

The preparation of stock solutions of PCBs at concentrations of 1 mg/L and 10 mg/L from liquid standards involved the mixing of PCB-Mix 3 and PCB-Mix 2 solutions in hexane in specific proportions. All stock solutions, along with intermediate standard solutions, were stored in 1 mL vials in a freezer at −20 °C. Their stability was monitored by examining the areas in the chromatograms monthly, and they were found to remain stable for a minimum of 6 months. The necessary solutions (1, 2, 5, 10, 25, 50, 100, 250, and 500 µg/L) used throughout the studies were freshly prepared each day through gradual dilutions with hexane in 1 mL vials. Additional information is available in the study by Güzel and Canlı [34].

#### 2.2.2. Sample Preparation and Analysis

A total of 0.5 g of dairy product or beverage sample was weighed and transferred into a clean extraction tube. IS solution (100 µL) and 4.5 mL of n-hexane were added to the sample to facilitate analyte extraction. For viscous and protein-rich matrices such as yogurt, kefir, and ayran, the samples were first subjected to centrifugation at 5000 rpm for 10 min to remove proteins and fat, and the supernatant was carefully collected. This pre-treatment step minimized clogging and ensured efficient loading onto solid-phase extraction (SPE) cartridges. After vortexing, the n-hexane extract was subjected to solid-phase extraction (SPE) for clean-up. Preconditioned SPE columns (SampliQ Silica and Silica SCX-Box), rinsed with 6 mL of n-hexane, were used to remove matrix interferences prior to instrumental analysis.

Following conditioning, 250 µL of the prepared sample extract was loaded onto the SPE cartridge. The elution step was carried out using 3 mL of n-hexane, to which 50 µL of tetradecane was added as a keeper. The eluate was then evaporated to near dryness under a gentle nitrogen stream and reconstituted to a final volume of 100 µL by first concentrating to 50 µL and then adding 50 µL of n-hexane. The final extract was analyzed by gas chromatography equipped with a tandem mass spectrometry detector (GC–MS/MS), using a 2 µL injection volume. In this study, PCBs levels are reported as µg/L wet weight.

#### 2.2.3. Analytical Instrumentation

The analysis of ICES-7 (International Council for the Exploration of the Sea) PCBs in dairy products and beverages was carried out using a Thermo Scientific Trace 1310 gas chromatography (GC) system (Waltham, MA, USA) coupled with a TSQ 8000 triple quadrupole mass spectrometer operating in Selected Reaction Monitoring (SRM) mode. Data acquisition and processing were conducted using Thermo TraceFinder EFS GC Analysis Software (Version 4.1, TSQ 8130309). Chromatographic separation was achieved on a TG-5SILMS fused silica capillary column (30 m × 0.25 mm i.d., 0.25 μm film thickness). An injection volume of 2 µL was used for all GC–MS/MS analyses. Detailed GC operating parameters for PCB analysis are provided in Table S3.

Table S4 provides comprehensive details on the GC–MS/MS operating conditions applied for PCB analysis, including retention times (RT), ionization mode, analyte-specific ions (target and precursor ions), and associated collision energies. Each PCB congener was confirmed in the mass spectrometer through two distinct transitions: a quantifying precursor ion (precursor ion-1) and a qualifying precursor ion (precursor ion-2), ensuring accurate identification and confirmation of the target compounds. During sample preparation, extracts were concentrated from the initial 4.5 mL solvent volume to a final reconstituted volume of 100 µL, corresponding to an overall concentration factor of approximately 45-fold.

#### 2.2.4. Quality Assurance/Quality Control (QA/QC)

Mass spectrometer auto-tuning on the GC–MSMS system was conducted using perfluorotributylamine (PFTBA) to ensure optimal sensitivity. Prior to sampling, all equipment was meticulously cleaned with acetone followed by hexane to prevent cross-contamination and identify any background signals during sampling runs. These solvent rinses were treated as control samples and underwent the same extraction procedures as the dairy product or beverage samples. In the control samples, only trace levels of PCB 28 were detected—less than 1% of the concentrations found in dairy product or beverage samples—while no other PCBs were present. The ideal reference material for matrix-matched quality control would be a certified pure milk standard. However, such certified reference materials were not commercially available during this study, and in-house preparation would not have provided the required standardization. Consequently, solvent-based blanks were employed strictly to monitor laboratory- and equipment-derived contamination, rather than to replicate matrix effects. Matrix-related variability was instead evaluated directly in the analyzed dairy and beverage samples, which represent the true biological matrices of interest. Once certified milk reference materials become available, future studies will be able to benefit from these materials, thus further strengthening method validation and comparability between laboratories.

Quality control measures were implemented in accordance with the standard analytical protocols for PCB determination. This included the analysis of laboratory blanks and IS recovery assessments for each sample. Laboratory blanks were prepared using n-hexane only (without dairy product or beverage matrices) and were subjected to the full extraction and clean-up procedures; no target analytes were detected in these blanks. ISs were added to all samples prior to extraction to assess the efficiency of the extraction and clean-up procedures. Recoveries of the target compounds were calculated based on the IS response. Furthermore, each PCB analyte was confirmed in the mass spectrometer by monitoring two specific ion transitions: a quantifying main ion/precursor ion-1 and a qualifying major ion/precursor ion-2 transition for additional confirmation.

The initial phase of the study focused on evaluating the method’s linearity, working range, and sensitivity, including the determination of the limits of detection (LOD) and quantification (LOQ) for measuring PCBs concentrations in the relevant samples. Table 1 presents detailed information on the calibration parameters for each PCB analyte, including the equations of the calibration curves (expressed as y = ax + b), correlation coefficients (R^2^), working ranges, and the corresponding LOD and LOQ values. The correlation coefficients of the calibration curves ranged from 0.9955 to 0.9997, indicating excellent linearity, with all R^2^ values exceeding 0.995. The LOD and LOQ values reported in Table 1 were determined using a combined instrumental and method-based approach: method LOD and LOQ were calculated according to the formulae (LOD = 3.3·σ/S; LOQ = 10·σ/S), where σ is the standard deviation of seven replicate analyses of low-level matrix-matched spiked samples and S is the slope of the matrix-matched calibration curve. Instrumental confirmation was performed on the GC–MS/MS chromatograms by verifying signal-to-noise ratios of ≥3 for LOD and ≥10 for LOQ using repeated injections of low-level standards. LOD/LOQ were therefore established based on calibration curve statistics and S/N evaluation, not from blank replicate analyses. Finally, the practical LOQ was set at the lowest concentration that fulfilled both the S/N criterion and acceptable method performance (70–120% recovery and RSD ≤ 20%).

**Figure 1:**
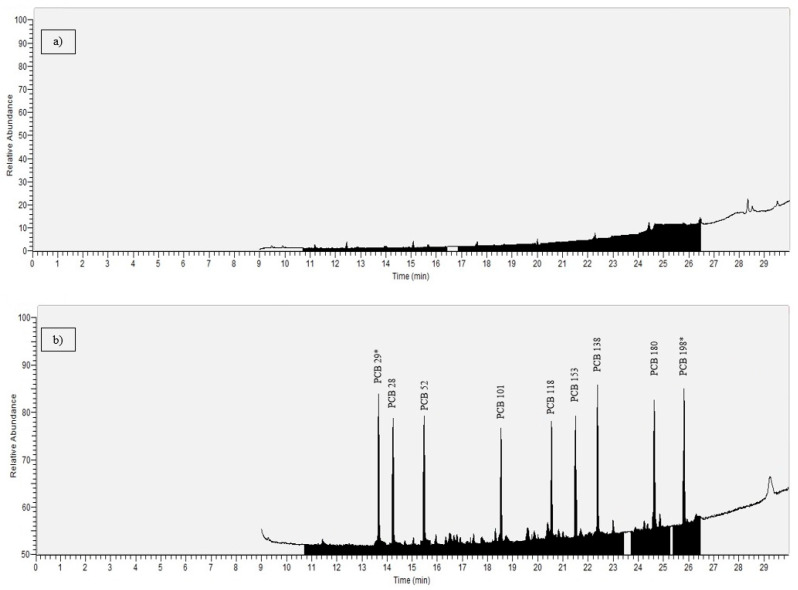
Representative GC–MS/MS chromatograms of ICES-7 PCBs: (**a**) blank sample showing no detectable PCB peaks, illustrating the baseline noise level, (**b**) matrix-matched samples spiked at the 1 µg/L, demonstrating clear and quantifiable peaks for ICES-7 PCBs and ISs are marked with *.

Representative chromatograms are provided in Figure 1, showing (a) a blank sample with no detectable PCB signals and (b) a sample spiked at the LOQ level, demonstrating clear and quantifiable peaks for all target analytes.

The recovery of ISs in spiked samples varied between 84.3% and 111.3%. Given that the average recovery exceeded 80%, all GC–MSMS results are reported without applying recovery correction factors. The IS concentrations in calibration solutions were consistent with those measured in the actual samples, supporting the validity of the quantification. Recoveries were evaluated using matrix-matched samples spiked at a concentration level of 25 µg/L (n = 7 replicates), which represents the mid-range of the calibration curve. LOD and LOQ values, used to assess the analytical sensitivity of the method, were calculated based on the average baseline noise in chromatograms, following procedures described in previous studies [35,36].

To evaluate the accuracy and precision of the analytical method, quality control (QC) analyses were conducted using standard solutions of PCB congeners at a nominal concentration of 250 ng/g. The results demonstrated acceptable levels of method performance in terms of both recovery and reproducibility (Table 2). The relative standard deviation (RSD) values for the analyzed congeners ranged between 3.18% and 9.94%, indicating good repeatability. Recovery rates varied from 91.5% (PCB 118) to 104.5% (PCB 180), all of which fall within the acceptable range (typically 70–120%) for trace-level analysis. Specifically, PCB 153 exhibited the highest analytical precision with an RSD of 3.18% and a recovery of 99.4%, while PCB 138 showed the highest RSD (9.94%) but still maintained a strong recovery of 96.3%. The average recovery across all PCB congeners was consistent and confirmed the method’s reliability without the need for correction factors. These findings support the robustness of the sample preparation and GC–MSMS quantification method applied in the study.

### 2.3. Health Risk Assessment

In this study, a comprehensive health risk assessment was performed to evaluate the potential adverse effects of consuming milk, dairy, and beverage products contaminated with PCBs. Key risk indicators—estimated daily intake (EDI) and hazard index (HI)—were used in the assessment (Table 3).

### 2.4. Data Analysis

Analyses were performed by transferring the study data to Microsoft Excel. For data evaluation, frequency distributions were calculated for categorical variables, and descriptive statistics (minimum, maximum, mean ± standard deviation) were provided for numerical variables. Three exposure scenarios—low, medium, and high—were considered to estimate average exposure from the consumption of milk, dairy products, and beverages. In the low-exposure scenario, values below the LOD and LOQ were assumed to be zero. In the medium scenario, values below the LOD and LOQ were replaced with LOD/2 and LOQ/2, respectively. In the high-exposure scenario, the LOD and LOQ values were treated as the actual concentrations.

## 3. Results and Discussions

### 3.1. Evaluation of PCBs Levels in Milk and Dairy Products

**Figure 2:**
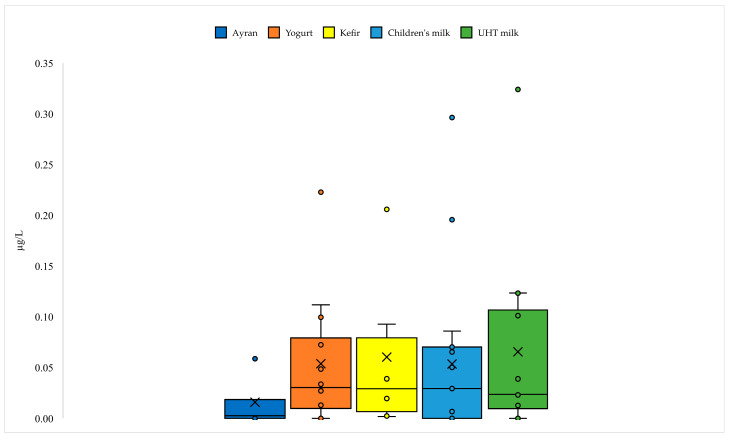
Distribution of ICES-7 levels in milk and dairy products.

ICES-7 levels in UHT milk, children’s milk, ayran, yogurt, and kefir samples were determined to not be detected (N.D.)–0.36, N.D.–0.32, N.D.–0.07, N.D.–0.23, and N.D.–0.22 µg/L wet weight, respectively (Figure 2). Overall, the PCBs levels detected in milk and dairy products were below the maximum level of 40 µg/kg set by the European Union for foodstuffs such as raw milk, dairy products, and meat products [40]. Therefore, all milk and dairy products examined were lower than the reference value.

In a study conducted in Italy, PCB6 levels in milk and yogurt samples were reported as 9.48 and 18.6 µg/L, respectively [41]. In a recent study conducted in Croatia, 534 milk and dairy products were examined, and PCBs were detected in only 5.23% of the samples. In this study, the average ICES-7 levels in cow, goat, dairy milk, and other dairy products were reported as 2.74 ± 5.42, 1.93 ± 0.18, 1.38 ± 0.48, and 2.85 ± 0.61 µg/kg, respectively, with the highest value (25.6 µg/kg) observed in cow’s milk [42]. In light of these data, the levels detected in the current study appear to be low compared to international averages. However, there are studies conducted in countries such as China, Bangladesh, the United States, and France that reported ICES-7 levels in milk and dairy products at levels lower than 0.5 µg/kg [43,44,45,46]. One of the main reasons for the discrepancy between this study and other studies in the literature is related to the variability of raw material type and content, production methods, and environmental conditions in the countries where the studies were conducted.

PCB 28, PCB 52, PCB 101, PCB 118, PCB 138, PCB 153, and PCB 180 levels in the examined milk and dairy products varied between N.D.–0.02, N.D.–0.03, N.D.–0.05, N.D.–0.05, N.D.–0.05, N.D.–0.09, and N.D.–0.13 µg/L wet weight, respectively (Table S5). As a result of the analyses, the detection frequencies of PCB 28, PCB 52, PCB 101, PCB 118, PCB 138, PCB 153, and PCB 180 in the examined milk and dairy products varied. Specifically, the concentrations of these PCBs in the samples were below the LOD/LOQ values in 48%, 48%, 48%, 80%, 44%, 39%, and 37% of the cases, respectively. These findings suggest that PCB 118 is the least common PCB congener in milk and dairy products, while PCB 153 and PCB 180 are more prevalent in the products.

Milk and dairy products were also ranked according to the maximum concentrations of each detected PCB (Table 4). The results indicate that UHT milk contains the highest concentrations for four different PCB congeners, while children’s milk exhibits the highest concentrations for three congeners. Ayran stands out as the product with the lowest contamination levels for all PCB congeners. Yogurt, with the exception of PCB 118, generally occupies a mid-range position in the ranking for all PCBs.

The findings reveal that PCBs contamination in milk and dairy products is generally low. While the levels of PCBs detected in kefir, UHT milk, and children’s milk are higher, PCB levels are generally lower in fermented products such as yogurt and ayran. The findings indicate that the levels of PCBs contamination in products are not constant, and that each PCB congener can be prominent in different products. For example, in UHT milk and children’s milk, particularly highly chlorinated PCB 153 and PCB 180 were detected at relatively higher levels than other PCBs. Conversely, lower molecular weight congeners such as PCB 28, PCB 52, and PCB 118 were found at lower levels in many products. Based on maximum ICES-7 levels, products are listed as UHT milk > children’s milk > yogurt > kefir > ayran. While products like ayran offer safer alternatives in terms of PCBs accumulation, kefir, UHT milk, and children’s milk carry a higher risk. Therefore, fermentation, low fat content, and production processes are thought to be factors limiting PCBs accumulation.

Products with a high fat content have a higher potential for accumulation due to the lipophilic nature of PCBs [47,48,49]. A similar pattern was observed in this study, where the levels of each PCB and ICES-7 were found to be higher in high-fat kefir, UHT milk, and children’s milk products compared to other products. The higher total PCB values found in directly and frequently consumed products such as UHT milk and children’s milk compared to others indicate the need for improved traceability of these product groups. The lower PCBs levels in fermented products (yogurt, ayran) can be explained by the fact that both the production process (fermentation) and chemical biotransformations limit the bioavailability of contaminants [50]. The generally lower fat content of these products may also be another important factor (Table S1). Indeed, these levels were found to be quite low in dilute, low-fat products like ayran. These products are typically consumed fresh due to their shorter shelf life, which reduces the duration of contact with packaging materials and may therefore limit PCBs migration and accumulation. The high PCB levels found in kefir, a fermented dairy product, were attributed to its higher fat content compared to ayran and yogurt (Table S1). However, since PCB levels were expressed on a fresh weight basis in this study, and the fat content in the samples could not be determined, this constitutes a significant limitation of the study. This study is limited by the expression of PCB levels on a fresh weight basis and the lack of fat content determination.

### 3.2. Evaluation of PCB Levels in Beverages

**Figure 3:**
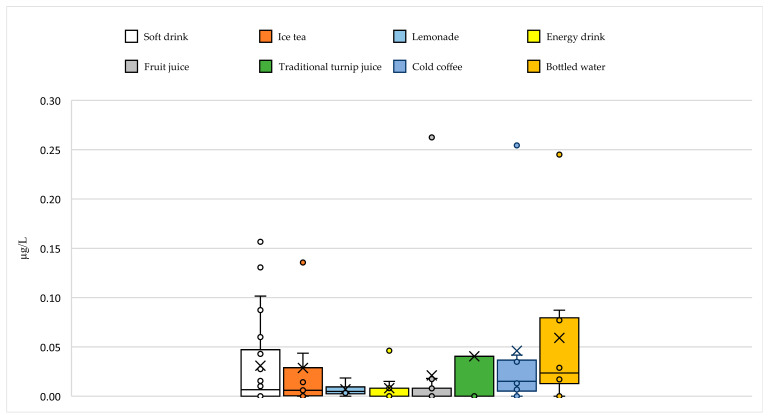
Distribution of ICES-7 levels in beverages.

ICES-7 levels in the examined beverage samples ranged from N.D. to 0.27 µg/L wet weight. ICES-7 levels in soft drink, lemonade, ice tea, energy drink, fruit juice, traditional turnip juice, cold coffee, and bottled water samples were determined as N.D.–0.17, N.D.–<0.02, N.D.–0.14, N.D.–0.03, N.D.–0.27, N.D.–0.17, N.D.–0.25, and N.D.–0.27 µg/L wet weight, respectively (Figure 3). All beverage samples examined were lower than the maximum level (40 µg/kg) set by the European Union for PCBs in certain foods [40].

As for international literature, a study conducted in Finland reported an average total level of PCBs of 0.08 µg/kg in coffee, tea, fruit juice, and other beverages [51]. A study conducted in Canada found no PCBs detected in tap and well water and tea samples [52]. In a comprehensive study conducted in Korea, total levels of PCBs were reported as 0.05–0.08 µg/kg in coffee samples, 0.07 µg/kg in mixed fruit juice samples, 0.07 µg/kg in green tea, 0.11 µg/kg in vegetable juice samples, 0.17 µg/kg in colada, and 0.12, 0.10, 0.07, 0.06, and 0.06 µg/kg in plum juice, tangerine juice, orange juice, grape juice, and tomato juice samples, respectively [53]. In a recent study conducted in Germany, the mean PCB6 level in coffee, cocoa, tea, and infusions was reported as 0.02 ± 0.03 (>0.00–0.08) µg/kg [48]. As observed in milk and dairy products, the discrepancies between the findings of this study and those reported in the literature can be attributed to variations in the chemical composition of raw materials, technological differences in production processes, and country-specific environmental factors where the studies were conducted (e.g., levels of industrial pollution, water and soil quality, and climatic conditions) [42,54,55,56].

In the examined beverage samples, PCB 28, PCB 52, PCB 101, PCB 118, PCB 138, PCB 153, and PCB 180 levels varied between N.D.–0.02, N.D.–0.03, N.D.–0.05, N.D.–0.05, N.D.–0.06, N.D.–0.08, and N.D.–0.08 µg/L wet weight, respectively (Table S6). As a result of the analyses, it was determined that these congeners in the beverage samples were below the LOD/LOQ value by 65% (PCB 28), 61% (PCB 52), 65% (PCB 101), 95% (PCB 118), 72% (PCB 138), 52% (PCB 153), and 56% (PCB 180), respectively. PCB contamination levels in beverage samples show a distribution similar to those obtained in milk and dairy products. Accordingly, the highest PCB levels in beverage samples were detected for PCB 180 and PCB 153, while the lowest were detected for PCB 118.

In the examined beverage samples, PCB 28, PCB 52, PCB 101, PCB 118, PCB 138, PCB 153, and PCB 180 levels varied between N.D.–0.02, N.D.–0.03, N.D.–0.05, N.D.–0.05, N.D.–0.06, N.D.–0.08, and N.D.–0.08 µg/L wet weight, respectively (Table S6). PCB contamination levels in beverage samples show a distribution similar to those obtained in milk and dairy products. Accordingly, the highest PCB levels in beverage samples were detected for PCB 180 and PCB 153, while the lowest were detected for PCB 118.

Based on the maximum concentrations of PCBs, fruit juice contained the highest levels of three different PCB congeners, traditional turnip juice contained two congeners, and bottled water contained one congener. Lemonade had the lowest contamination level of all PCB congeners. Soft drink samples generally exhibited a moderate contamination profile across all PCB congeners (Table 5).

These findings indicate that PCBs contamination in the beverage samples evaluated in this study was generally low. According to the maximum ICES-7 value, beverages were ranked as follows: fruit juice = bottled water > cold coffee > soft drink = traditional turnip juice > ice tea > energy drink > lemonade. This ranking indicates that some structural and content-based trends (e.g., fat content, production method, use of additives, etc.) may be effective in determining maximum PCBs levels in beverages.

The high ICES-7 levels detected in bottled water may be indicative of contamination through migration from filling systems or packaging materials, as well as environmental contaminants that may be present in the drinking water source. Furthermore, the milk or vegetable oil additives used in cold coffee samples, which detected high ICES-7 levels, could be considered matrices that facilitate PCBs migration. The high levels of PCBs detected in fruit juice samples could be explained by the relatively more natural structure of these products and the natural lipid content of these samples, which contribute to the accumulation of PCBs. Conversely, in samples such as soft drinks and ice tea, which contain moderate levels of ICES-7, additives added to the ingredients or packaging materials used in the production process could be considered potential sources of contamination. The ICES-7 values detected in turnip juice, a traditional fermented beverage, were similar to those in other carbonated and non-fermented beverages. However, the fact that the product in question, like other fruit juice samples, had a natural structure and contained lipid derivatives was a factor that increased the likelihood of detecting high PCBs levels in this group. However, the findings suggest that the fermentation process does not increase PCBs accumulation; on the contrary, it may play a role in reducing bioavailability under some conditions. This hypothesis is also supported by the low PCBs levels detected in milk-based fermented products such as ayran and yogurt. In this context, the microbiological properties of fermented products represent an important research area for investigating the effects on the bioavailability and metabolism of PCBs. The low PCBs levels found in samples such as lemonade and energy drinks indicate that these products do not provide a suitable environment for PCBs migration. This suggests that the chemical composition of the beverage matrix plays a critical role in PCBs accumulation. This finding also indicates that additives and industrial flavorings are not direct sources of PCBs, and that the risk is more related to the raw material source, the product’s lipid content, the production process (e.g., fermentation), and packaging conditions. Indeed, Shin et al. linked low PCBs levels in beverages to high water content and low lipid content [53]. In addition, controlled water sources and industrial processing techniques used in beverage production contribute to low contamination levels in commercial beverages.

### 3.3. Evaluation of PCBs Levels According to Packaging Types

**Figure 4:**
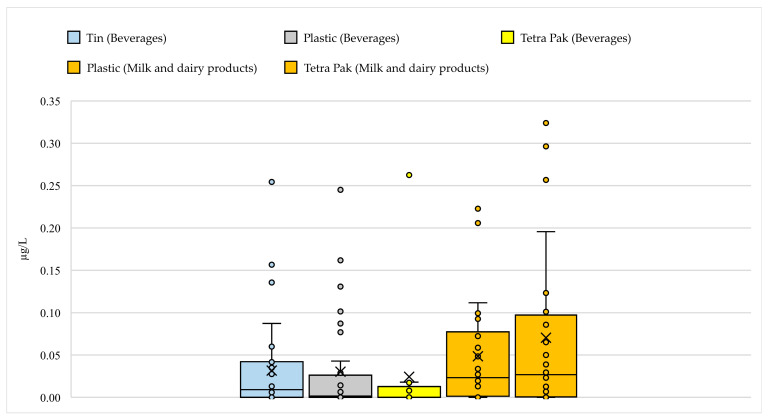
Distribution of ICES-7 levels according to packaging types.

Within the scope of the study, different packaging types used for beverages and milk and dairy products were comparatively evaluated in terms of PCBs contamination levels. ICES-7 levels in tin, plastic, and Tetra Pak packages in the beverage category were generally similar, ranging from N.D.–0.26, N.D.–0.29, and N.D.–0.26 µg/L wet weight, respectively. In the milk and dairy products category, ICES-7 levels in plastic packages were N.D.–0.26 µg/L wet weight, while this value was N.D.–0.42 µg/L wet weight in Tetra Pak packages (Figure 4). When comparing similar packages, it can be said that the ICES-7 levels detected in plastic packages were similar in both product groups. However, it is noteworthy that the ICES-7 levels detected in Tetra Pak packages (milk and dairy products) were significantly higher than those in beverages packaged in the same packaging type. This difference is thought to be due to both the chemical properties of the product matrix, such as its fat content, and the composition or manufacturing process of the packaging material. As seen in the example of Tetra Pak packaging, the chemical structure of the product matrix (e.g., water-based beverages vs. fat-based dairy products) is a determining factor in PCBs contamination, even when the packaging type is the same. It should also be considered that packaging materials containing recycled content may increase the risk of migration of lipophilic contaminants such as PCBs. This data is important for the selection and monitoring of packaging materials. It should be noted that mixed-structure packaging, such as Tetra Pak, can be a potential vector for PCBs contamination.

One of the striking findings of the study is the difference in the prevalence of PCB congeners across packaging types. For the beverage group, maximum levels of PCB 101, PCB 153, and PCB 180 were relatively higher in Tetra Pak packages, maximum levels of PCB 52 and PCB 138 in plastic packages, and maximum levels of PCB 118 in tin packages, compared to other packaging types. In the milk and dairy products group, levels of all PCB congeners except PCB 118 were higher in Tetra Pak-packaged products than in plastic-packaged products (Table S7). This suggests that PCBs contamination varies across packaging types in both product groups and suggests that packaging materials should be considered as potential sources of contamination. Each packaging material may exhibit different permeability and adsorption properties toward specific PCB congeners depending on factors such as its chemical structure, production process, recycling history, and duration of contact with food. For example, as plastic packaging is generally made from low-permeability polymers, it may pose a limited risk of contamination [57]. These compounds can migrate into products through additives used during packaging production or equipment in contact with the plastic [58,59]. Tetra Pak packaging, on the other hand, is a multilayered structure composed of polyethylene, aluminum, and cardboard layers [60]. This complex structure can increase the risk of both permeability to external environmental contaminants and migration from the packaging’s own components. The polyethylene layer’s potential to absorb or transfer lipophilic contaminants, such as PCBs, can be a source of contamination. Tin cans are often coated with an epoxy coating on their inner surfaces to prevent direct metal contact with food [61]. However, these coatings carry a risk of contamination with various contaminants depending on the production process [62]. Additionally, it should be noted that factors such as filling at high temperatures, storage time, and the chemical composition of the can’s inner coating can affect the level of contamination [63]. Based on these findings, it can be concluded that each packaging type has a specific risk profile against specific PCB congeners. Therefore, a more accurate and scientific approach would be to focus not only on total PCB levels in food packaging safety assessments, but also on the profiles of individual congeners and how these profiles vary by packaging type.

### 3.4. Dietary PCBs Exposure

Although the PCBs levels detected in milk and dairy products, and beverages in this study, were low, this does not mean that potential health risks can be completely ruled out. Exposure to PCBs in food is a chronic process that continues throughout life, and the long-term effects of PCBs, particularly on the endocrine, neurological, and immunological systems, are still under scientific debate. This chronic exposure can pose cumulative health risks, particularly for developing children, pregnant women, and immunocompromised individuals. Therefore, the study evaluated PCB exposure levels and non-carcinogenic health risks from milk, dairy products, and beverage consumption for adults and children under three different scenarios (low, medium, and high).

#### 3.4.1. PCBs Exposure Level from Consumption of Milk and Dairy Products

In estimating PCB exposure levels from the consumption of milk and dairy products, the average PCBs concentrations detected in each product were used as the reference values for the low, medium, and high scenarios. The average ICES-7 exposure levels of individuals from the consumption of milk and dairy products are shown in Table 6. The highest and lowest daily ICES-7 exposure levels for adults arise from children’s milk and ayran consumption. The daily ICES-7 exposure levels for adults resulting from consumption of UHT milk and yogurt are in the range of 0.19 ± 0.27–0.21 ± 0.26 and 0.10 ± 0.13–0.12 ± 0.13 ng/kg/day for the three scenarios, respectively. The World Health Organization (WHO) proposed a tolerable daily intake (TDI) of 20 ng/kg/day for total PCBs in 2003 [37]. In the high exposure scenario, the contribution of ICES-7 exposure from the consumption of milk and dairy products to the TDI was calculated as 0.40–3.30%. In line with this value, the ICES-7 exposure levels per body weight resulting from consumption of milk and dairy products examined in the study remain well below the TDI in all scenarios.

The level of exposure is directly related to the PCBs concentrations in the product and the amount consumed. In this context, although the average ICES-7 level calculated for kefir across all scenarios was lower than that of UHT milk, the single-use packaging volume in which this product is commercially sold was approximately 50% higher than that of other products (Table S1). Therefore, the amount of kefir consumed directly affected the exposure level. UHT milk, on the other hand, has the highest concentration of ICES-7, and its widespread consumption in the daily diet places this product at higher risk for PCBs exposure. The mean ICES-7 level calculated for children’s milk was the highest across all scenarios, reflecting both the generally higher concentrations in children’s milk compared to buttermilk and yogurt, and the substantially lower body weight of children relative to adults. This clearly demonstrates that children are a more vulnerable group to PCBs exposure.

Sirot et al. [43] reported the average PCB6 exposure level from consumption of milk and other dairy products as 0.05 and 0.10 ng/kg/day for adults and 0.23 and 0.22 ng/kg/day for children, respectively. The same study calculated the contribution of milk and dairy product consumption to daily PCB6 exposure as 1.6–3.5% and 5.8–5.9% for adults and children, respectively [43]. Hasan et al. reported the PCB4 exposure level from cow’s milk consumption as 3.97 ng/kg/day for adults [46]. Barone et al. [41] reported the PCB6 exposure level from milk and dairy products consumption as 3.18 ng/kg/day for adults. In the same study, the contribution of milk and dairy products to daily PCB6 exposure level was reported as 27.8% [41]. The daily ICES-7 exposure levels for adults and children through the consumption of milk and dairy products have been reported as 5.59 and 18.2 ng/kg/day, respectively, which are considerably higher than the values observed in the present study [42]. Poland and Austria reported exposure levels for adults as 2.1 and 1.34–1.74 ng/kg/day, respectively [64,65]. Therefore, the ICES-7 exposure levels calculated based on milk and dairy products consumption in this study are higher compared to the values reported by Sirot et al. [43], but lower compared to other international studies. Differences in PCB exposure levels reported in different studies are due to the fact that the PCBs contents in milk and dairy products analyzed vary depending on the study, as well as the frequency and amount of consumption of these products vary significantly between countries.

#### 3.4.2. PCBs Exposure Level from Consumption of Beverages

In calculating PCBs exposure levels from beverage consumption, the average PCBs concentrations detected in each product were used as the reference values for the low, medium, and high scenarios. The average ICES-7 exposure levels of individuals from the consumption of beverages are shown in Table 6. ICES-7 exposure levels from beverage consumption ranged from 0.03 ± 0.04 to 0.20 ± 0.41, 0.05 ± 0.03 to 0.21 ± 0.40, and 0.07 ± 0.03 to 0.23 ± 0.40 ng/kg/day for the low, medium, and high scenarios, respectively. The contribution of ICES-7 exposure levels from beverage consumption to the TDI was calculated to be a maximum of 1.12% for all scenarios. ICES-7 exposure levels for all beverages in three different scenarios are significantly lower than the TDI value (20 ng/kg/day) [37].

The beverages with the highest daily ICES-7 exposure levels for adults are cold coffee, bottled water, and ice tea/soft drink, respectively, while the beverages with the lowest levels are lemonade, fruit juice, and energy drinks. As with milk and dairy products, this ranking was influenced by the PCB levels detected in the products and the amount consumed. While the highest average ICES-7 levels calculated for all scenarios were found in bottled water samples, the higher consumption of cold coffee products compared to bottled water changed the exposure ranking. However, it should be noted that this finding is only valid for assessments based on single-use packaging volumes. A much higher total daily water consumption compared to other beverages may increase the impact of water on PCBs exposure in real-life conditions. This suggests that consumption scenarios can alter the exposure ranking.

Studies examining PCB exposure levels from beverage consumption are limited. Shin et al. estimated the contribution of PCBs exposure from beverage consumption to adult daily PCBs intake in Korea as 5.19% and reported it as 0.20 ng/kg/day [53]. The variations in PCB exposure levels reported across studies concerning beverages can be attributed to differences in the concentrations of PCBs detected in the analyzed samples, as well as to the variability in beverage consumption habits across countries. Therefore, interpreting PCBs exposure data from beverages requires careful consideration of both product-specific contamination and population-specific consumption patterns.

#### 3.4.3. PCB Exposure Levels According to Product Packaging

Within the scope of the study, PCBs exposure was also evaluated according to product packaging. The average ICES-7 exposure levels of individuals resulting from product consumption by packaging type are presented in Table 7.

The ranking of exposure levels for beverage packaging across all scenarios is tin > plastic > Tetra Pak, while the ranking for milk and dairy products packaging across all scenarios is Tetra Pak > plastic. When both groups were evaluated individually, the consumption volume of the packaging and the PCB levels in the product were the determining factors. However, when comparing similar packaging in both groups, the PCB levels of the product were the determining factor. For example, the fruit juice packaged in Tetra Pak in the beverage group and the children’s milk and UHT milk packaged in the milk and dairy products group had almost the same packaging volume (200 mL). However, the average daily ICES-7 exposure from Tetra Pak-packaged milk and dairy products was approximately five times higher than that of similar packaging in the beverage group. A similar situation also applies to plastic packaging in both product groups.

### 3.5. Non-Carcinogenic Health Risk Assessment

Although the PCBs levels detected in the milk, dairy products, and beverage samples examined in this study were found to be low, it is clear that these products are frequently consumed in daily life and that the potential health risks of PCBs should be carefully evaluated. In this context, HI values were determined from ICES-7 levels, taking into account three exposure scenarios for each sample and packaging type (Table 8 and Table 9).

The results presented in Table 8 and Table 9 indicate that the HI values calculated for ICES-7 across all sample groups—including milk and dairy products, beverages, and various packaging types—remained well below the threshold value of 1. This suggests that, under the evaluated exposure scenarios, the non-carcinogenic health risks associated with PCBs intake through consumption of these products are negligible. Many studies have reported that HI values for ICES-7 exposure levels resulting from milk, dairy products, and beverage consumption are lower than reference values and that the PCBs levels in these products are reliable in terms of non-carcinogenic health risks [49,53,65]. However, there are also studies reporting HI values for ICES-7 exposure levels resulting from the milk and dairy products consumption of adults and children as 0.28 and 0.91, respectively, which are close to the reference values [42].

The low HI values (<1) may be attributed to regulatory improvements in food safety, increased monitoring of persistent organic pollutants, and advances in packaging technologies that minimize contamination. It is also important to note that dairy and beverage products typically undergo standardized processing and quality control steps, which may further reduce PCBs presence. However, despite these reassuring results, cumulative exposure over a lifetime and combined effects with other environmental contaminants should not be overlooked. Future studies should consider long-term dietary exposure assessments, vulnerable population groups (such as children or pregnant individuals), and potential synergistic effects of multiple pollutants. In this context, the current data supports the safety of the tested products, but continued surveillance and broader ecological risk assessments remain essential to ensure public health protection.

## 4. Conclusions

This study provides a systematic evaluation of PCB contamination levels in milk, dairy products, and various beverages in Türkiye, with comprehensive assessment of dietary exposure patterns and non-carcinogenic health risks across population subgroups. Using a rigorous analytical approach, samples were evaluated across three consumption scenarios (low, medium, high) for both adults and children, enabling robust exposure estimation. While overall contamination levels were low, higher ICES-7 concentrations were detected in high-fat products such as UHT milk and children’s milk. In contrast, fermented dairy products like yogurt, kefir, and ayran showed lower PCBs levels, suggesting a possible mitigating effect of low fat content and fermentation. Among beverages, a clear matrix-dependent pattern emerged, with fat-containing or complex-matrix products (e.g., cold coffee) demonstrating 2–3 times higher PCB levels than fat-free or additive-based drinks like lemonade and energy drinks. Differences in PCB contamination levels were also detected based on packaging type. Tetra Pak-packaged milk and dairy products, in particular, had the highest PCB levels. This indicates that both packaging material and product matrix play a role in PCBs migration and accumulation. Health risk assessments revealed that all HI values remained well below the safety threshold of 1, suggesting negligible non-carcinogenic risk. However, cumulative exposure—especially for children and sensitive populations—warrants attention. The findings highlight the importance of monitoring not only production and processing stages but also packaging and product composition in controlling persistent organic pollutants. In conclusion, addressing PCBs contamination in dairy products requires strengthening regulatory limits and enforcement, implementing regular monitoring programs, and improving quality control along the production chain. To control PCB levels in animal-derived foods, feed sources should be continuously monitored, and the use of contaminated feed strictly avoided. Products with high fat content, in particular, should be analyzed regularly due to their higher risk of contaminant accumulation. Furthermore, the development of rapid detection methods and effective risk communication for vulnerable groups may help reduce exposure and better protect public health.

## Figures and Tables

**Table 1:** Linearity, operating range, LOD, LOQ data for PCBs.

Analytes	R^2^	Calibration Curve Equation (y = ax + b)	Working Range (µg/L)	LOD (µg/L)	LOQ (µg/L)	RSD (%)
PCBs						
PCB 101	0.9967	y = 8691x + 43.606	1–500	0.001	0.003	7.12
PCB 118	0.9988	y = 20,290x − 8183.6	1–500	0.001	0.003	4.48
PCB 153	0.9975	y = 5546.3x + 33.861	1–500	0.001	0.003	3.01
PCB 138	0.9986	y = 6609x + 39.380	1–500	0.001	0.003	4.17
PCB 180	0.9970	y = 2781.6x + 15.058	1–500	0.001	0.003	5.11
PCB 198 *	-	-	25	-	-	6.19
PCB 28	0.9955	y = 55.488x + 255.461	1–500	0.001	0.003	5.87
PCB 29 *	-	-	25	-	-	6.66
PCB 52	0.9997	y = 10.557x + 33.457	1–500	0.001	0.003	6.55

* Internal standard.

**Table 2:** The results of all PCBs in the quality control study.

Analytes	Quality Control Conc. (µg/kg)	Measured Conc. (µg/L)	RSD (%)	Recovery (%)
PCB 101	250	239.2	7.15	95.7
PCB 118	250	228.7	3.34	91.5
PCB 153	250	248.4	3.18	99.4
PCB 138	250	240.7	9.94	96.3
PCB 180	250	261.2	4.04	104.5
PCB 101	250	255.7	5.15	102.3
PCB 28	250	229.9	8.21	92.0
PCB 52	250	235.5	4.18	94.2

**Table 3:** Health risk assessment formula, variables, and criteria.

Cutoff Point	Formula	Variables	Criteria
Estimated Daily Intake (EDI) (ng/kg/day)	$\mathrm{EDI}=\frac{C\;\times \;\mathrm{IR}}{\mathrm{bw}\;\times \;1000}$	C is the concentration of each PCB in each sample (ng/L), IR is the intake rate of each sample (mL/day), bw is the body weight (kg), and 1000 is the conversion factor. The analyzed milk, dairy, and beverage products are marketed as single-use items; therefore, the consumption amount was assumed to be equivalent to the portion size indicated on the packaging (Tables S1 and S2). The body weights of children and adults were assumed to be 17.5 and 70 kg, respectively.	ICES-7 exposure levels should not exceed the maximum reference value (20 ng/kg/day) [37].
Hazard Index (HI) (It has no units)	$\mathrm{HI}=\sum_{i=1}^{7}{\mathrm{THQ}}_{1}=\frac{\mathrm{EDI}}{\mathrm{RfD}}$	Target Hazard Quotient (THQ). The RfD is the oral reference dose (ng/kg/day). The RfD values of PCB 28, PCB 52, PCB 101, PCB 118, PCB 138, PCB 153, and PCB 180 were determined as 20 ng/kg/day [38].	While THQ indicates a significant health problem that is not carcinogenic, THQ <1 means that there is insignificant risk about health hazard [39].

**Table 4:** Ranking of milk and dairy products based on the maximum concentrations of PCBs.

PCB 28	UHT milk = children’s milk = yogurt = kefir = ayran
PCB 52	Children’s milk > UHT milk > kefir = yogurt = ayran
PCB 101	Children’s milk > UHT milk > kefir > yogurt = ayran
PCB 118	Yogurt > UHT milk = kefir > children’s milk > ayran
PCB 138	UHT milk > children’s milk >yogurt = kefir > ayran
PCB 153	UHT milk > children’s milk > kefir > yogurt > ayran
PCB 180	UHT milk > children’s milk > yogurt = kefir > ayran

**Table 5:** Ranking of beverages based on the maximum concentrations of PCBs.

PCB 28	Traditional turnip juice = cold coffee > fruit juice = bottled water = soft drink = ice tea = energy drink > lemonade
PCB 52	Traditional turnip juice > fruit juice = bottled water = cold coffee = soft drink > ice tea > energy drink = lemonade
PCB 101	Fruit juice > bottled water = traditional turnip juice> soft drink > cold coffee = ice tea > energy drink = lemonade
PCB 118	Cold coffee > bottled water > ice tea > soft drink = fruit juice= traditional turnip juice= energy drink = lemonade
PCB 138	Bottled water > fruit juice = cold coffee > soft drink = ice tea = traditional turnip juice > energy drink > lemonade
PCB 153	Fruit juice > bottled water = cold coffee > soft drink > ice tea = traditional turnip juice > energy drink > lemonade
PCB 180	Fruit juice > bottled water > cold coffee = soft drink > ice tea > traditional turnip juice > energy drink > lemonade

**Table 6:** Daily mean ICES-7 exposure levels from the consumption of milk, dairy products, and beverages (ng/kg/day).

Products	Low (mean ± SD; min–max)	Medium (mean ± SD; min–max)	High (mean ± SD; min–max)
Milk and dairy products			
UHT Milk	0.19 ± 0.27 (0–0.93)	0.20 ± 0.27 (0.01–0.93)	0.21 ± 0.26 (0.02–0.93)
Children’s milk	0.61 ± 0.92 (0–3.39)	0.63 ± 0.91 (0.04–3.39)	0.65 ± 0.90 (0.08–3.39)
Ayran	0.05 ± 0.08 (0–0.17)	0.06 ± 0.08 (0.01–0.17)	0.07 ± 0.07 (0.02–0.17)
Yogurt	0.10 ± 0.13 (0–0.40)	0.11 ± 0.13 (0.01–0.40)	0.12 ± 0.13 (0.01–0.40)
Kefir	0.23 ± 0.28 (0–0.74)	0.24 ± 0.28 (0.01–0.74)	0.25 ± 0.27 (0.02–0.74)
Beverages			
Soft drink	0.15 ± 0.22 (0–0.74)	0.17 ± 0.20 (0.02–0.74)	0.19 ± 0.20 (0.03–0.74)
Lemonade	0.03 ± 0.04 (0–0.09)	0.05 ± 0.03 (0.02–0.09)	0.07 ± 0.03 (0.03–0.09)
Ice tea	0.14 ± 0.23 (0–0.64)	0.15 ± 0.23 (0.02–0.64)	0.17 ± 0.22 (0.03–0.64)
Energy drink	0.04 ± 0.07 (0–0.22)	0.06 ± 0.07 (0.02–0.22)	0.08 ± 0.06 (0.03–0.22)
Fruit juice	0.06 ± 0.20 (0–0.75)	0.07 ± 0.19 (0.01–0.75)	0.09 ± 0.18 (0.02–0.75)
Traditional turnip juice	0.12 ± 0.23 (0–0.46)	0.13 ± 0.22 (0.01–0.46)	0.14 ± 0.22 (0.02–0.46)
Cold coffee	0.20 ± 0.41 (0–1.19)	0.21 ± 0.40 (0.02–1.19)	0.23 ± 0.40 (0.03–1.19)
Bottled water	0.17 ± 0.23 (0–0.70)	0.18 ± 0.23 (0.02–0.70)	0.19 ± 0.22 (0.04–0.70)

**Table 7:** Daily mean ICES-7 exposure levels by packaging type for milk, dairy products, and beverages (ng/kg/day).

Products	Low	Medium	High
Milk and dairy products			
Plastic	0.13 ± 0.18 (0–0.74)	0.14 ± 0.18 (0.01–0.74)	0.15 ± 0.17 (0.01–0.74)
Tetra Pak	0.45 ± 0.72 (0–3.39)	0.46 ± 0.72 (0.01–3.39)	0.48 ± 0.71 (0.02–3.39)
Beverages			
Tin	0.14 ± 0.26 (0–1.19)	0.16 ± 0.25 (0.01–1.19)	0.18 ± 0.24 (0.03–1.19)
Plastic	0.11 ± 0.19 (0–0.70)	0.12 ± 0.18 (0.01–0.70)	0.14 ± 0.18 (0.02–0.70)
Tetra Pak	0.07 ± 0.21 (0–0.75)	0.08 ± 0.20 (0.01–0.75)	0.10 ± 0.20 (0.02–0.75)

**Table 8:** HI values for ICES-7 in milk, dairy, and beverage products (mean ± SD; min–max).

Products	Low (mean ± SD; min–max)	Medium (mean ± SD; min–max)	High (mean ± SD; min–max)
Milk and dairy products			
UHT Milk	0.01 ± 0.01 (0–0.05)	0.01 ± 0.01 (<0.01–0.05)	0.01 ± 0.01 (<0.01–0.05)
Children’s milk	0.03 ± 0.05 (0–0.19)	0.03 ± 0.05 (<0.01–0.19)	0.03 ± 0.05 (<0.01–0.19)
Ayran	<0.01 ± 0.00 (0–0.01)	<0.01 ± 0.00 (<0.01–0.01)	<0.01 ± 0.00 (<0.01–0.01)
Yogurt	0.01 ± 0.01 (0–0.02)	0.01 ± 0.01 (<0.01–0.02)	0.01 ± 0.01 (<0.01–0.02)
Kefir	0.01 ± 0.01 (0–0.04)	0.01 ± 0.01 (<0.01–0.04)	<0.01 ± 0.01 (<0.01–0.04)
Beverages			
Soft drink	0.01 ± 0.01 (0–0.02)	0.01 ± 0.01 (<0.01–0.04)	0.01 ± 0.01 (<0.01–0.04)
Lemonade	<0.01 ± 0.00 (0–0.01)	<0.01 ± 0.00 (<0.01–0.01)	<0.01 ± 0.00 (<0.01–0.01)
Ice tea	0.01 ± 0.01 (0–0.03)	0.01 ± 0.01 (<0.01–0.03)	0.01 ± 0.01 (<0.01–0.03)
Energy drink	<0.01 ± 0.00 (0–0.01)	<0.01 ± 0.00 (<0.01–0.01)	<0.01 ± 0.00 (<0.01–0.01)
Fruit juice	<0.01 ± 0.01 (0–0.04)	<0.01 ± 0.01 (<0.01–0.04)	0.01 ± 0.01 (<0.01–0.04)
Traditional turnip juice	0.01 ± 0.01 (0–0.02)	0.01 ± 0.01 (<0.01–0.02)	0.01 ± 0.01 (<0.01–0.02)
Cold coffee	0.01 ± 0.02 (0–0.06)	0.011 ± 0.02 (<0.01–0.061)	0.01 ± 0.02 (<0.01–0.06)
Bottled water	0.01 ± 0.01 (0–0.04)	0.01 ± 0.01 (<0.01–0.04)	0.01 ± 0.01 (<0.01–0.04)

**Table 9:** HI values for ICES-7 in packaging types (mean ± SD; min–max).

Products	Low	Medium	High
Milk and dairy products			
Plastic	0.01 ± 0.01 (0–0.04)	0.01 ± 0.01 (<0.01–0.04)	0.01 ± 0.01 (<0.01–0.04)
Tetra Pak	0.02 ± 0.04 (0–0.19)	0.02 ± 0.04 (<0.01–0.19)	0.03 ± 0.04 (<0.01–0.19)
Beverages			
Tin	0.01 ± 0.01 (0–0.06)	0.01 ± 0.01 (<0.01–0.06)	0.01 ± 0.01 (<0.01–0.06)
Plastic	0.01 ± 0.01 (0–0.04)	0.01 ± 0.01 (<0.01–0.04)	0.01 ± 0.01 (<0.01–0.04)
Tetra Pak	<0.01 ± 0.01 (0–0.04)	<0.01 ± 0.01 (<0.01–0.04)	0.01 ± 0.01 (<0.01–0.04)

## Data Availability

All data are described in the article.

## Supplementary Materials

The following supporting information can be downloaded at: https://www.mdpi.com/article/10.3390/foods14203544/s1, Table S1: Some information about the dairy product samples; Table S2: Some information about the beverage samples; Table S3: GC analysis program for PCBs; Table S4: GC–MSMS method parameters for each PCB analytes; Table S5: PCBs levels in milk and dairy products (mean ± SD; min–max) (µg/L wet weight); Table S6: PCBs levels in beverages (mean ± SD; min–max) (µg/L wet weight); Table S7: PCBs levels in packaging types (mean ± SD; min–max) (µg/L wet weight).
