# Supplementary material for: Monitoring of Polychlorinated Biphenyls (Pcbs) Contamination in Milk and Dairy Products and Beverages in Türkiye: A Public Health Perspective
Source: Foods. 2025 Oct 17;14(20):3544. doi: 10.3390/foods14203544 (PMC12562641; doi:10.3390/foods14203544)
Supplement: Supplementary file 1 [file foods-14-03544-s001.zip › foods-3859230-supplementary.pdf]

Monitoring of polychlorinated biphenyls (PCBs) contamination in milk and dairy products and beverages in Türkiye: A public health perspective

Oltan Canlı[1], Barış Güzel[1], Merve Türk[1,2], Burhan Basaran[3]*

[1] Climate Studies and Water Management Research Group, Climate and Life Vice Presidency, TUBITAK Marmara Research Center, 41470, Gebze, Kocaeli, Türkiye.

[2] Kocaeli University, Environmental Engineering, Umuttepe Campus, 41275, Izmit, Kocaeli, Türkiye.

[3] Department of Nutrition and Dietetics, Faculty of Health Sciences, Recep Tayyip Erdogan University, Rize, 53100, Türkiye.

* Corresponding Author e-mail: burhan.basaran@erdogan.edu.tr; simge.sipahi@acibadem.edu.tr

Table S1. Some information about the dairy product samples

Sample No	Packaging type	Product features	Consumption amount	Fat content according to label information
Sample 1	Plastic	Buttermilk	170 mL	0.5%
Sample 2	Plastic	Buttermilk	200 mL	0.5%
Sample 3	Plastic	Buttermilk	200 mL	0.5%
Sample 4	Plastic	Buttermilk	200 mL	0.5%
Sample 5	Plastic	Yogurt	200 g	3.5%
Sample 6	Plastic	Yogurt	200 g	1.4%
Sample 7	Plastic	Yogurt	200 g	2.2%
Sample 8	Plastic	Yogurt	200 g	1.4%
Sample 9	Plastic	Yogurt (fruit)	67 g	1.0%
Sample 10	Plastic	Yogurt (strawberry)	65 g	2.2%
Sample 11	Plastic	Yogurt (strawberry)	90 g	1.8%
Sample 12	Plastic	Yogurt (strawberry)	125 g	1.4%
Sample 13	Plastic	Yogurt (strawberry-probiotic)	100 g	2.1%
Sample 14	Plastic	Yogurt (fruit)	90 g	1.4%
Sample 15	Plastic	Yogurt (probiotic)	100 g	1.9%
Sample 16	Plastic	Yogurt (fruit)	125 g	2.1%
Sample 17	Plastic	Kefir (strawberry)	330 mL	2.3%
Sample 18	Plastic	Kefir	330 mL	3.0%
Sample 19	Plastic	Kefir	330 mL	2.7%
Sample 20	Plastic	Kefir (strawberry)	200 mL	2.7%
Sample 21	Plastic	Kefir	250 mL	2.1%
Sample 22	Plastic	Kefir	290 mL	3.0%
Sample 23	Tetra Pak	Children's milk (strawberry)	180 mL	1.2%
Sample 24	Tetra Pak	Children's milk (strawberry)	180 mL	1.2%
Sample 25	Tetra Pak	Children's milk (strawberry)	200 mL	1.5%
Sample 26	Tetra Pak	Children's milk	330 mL	1.5%
Sample 27	Tetra Pak	Children's milk (banana)	180 mL	1.2%
Sample 28	Tetra Pak	Children's milk (banana)	200 mL	1.8%
Sample 29	Tetra Pak	Children's milk (banana)	200 mL	1.5%
Sample 30	Tetra Pak	Children's milk (cocoa)	180 mL	1.5%
Sample 31	Tetra Pak	Children's milk (banana)	180 mL	1.2%
Sample 32	Tetra Pak	Children's milk (banana)	200 mL	1.8%
Sample 33	Tetra Pak	Children's milk (banana)	180 mL	1.2%
Sample 34	Tetra Pak	Children's milk (cocoa)	200 mL	1.2%
Sample 35	Tetra Pak	Children's milk (cocoa)	180 mL	1.5%
Sample 36	Tetra Pak	Children's milk (cocoa)	180 mL	1.5%
Sample 37	Tetra Pak	Children's milk (cocoa)	200 mL	1.2%
Sample 38	Tetra Pak	Children's milk (cocoa)	180 mL	1.8%
Sample 39	Tetra Pak	Children's milk (cocoa)	200 mL	1.2%
Sample 40	Tetra Pak	UHT Milk	200 mL	3.5%
Sample 41	Tetra Pak	UHT Milk	200 mL	2.5%
Sample 42	Tetra Pak	UHT Milk	200 mL	1.5%
Sample 43	Tetra Pak	UHT Milk	200 mL	3.5%
Sample 44	Tetra Pak	UHT Milk	200 mL	3.5%
Sample 45	Tetra Pak	UHT Milk	200 mL	1.5%
Sample 46	Tetra Pak	UHT Milk	200 mL	3.5%
Sample 47	Tetra Pak	UHT Milk	200 mL	2.5%
Sample 48	Tetra Pak	UHT Milk	200 mL	3.5%
Sample 49	Tetra Pak	UHT Milk	200 mL	1.5%
Sample 50	Tetra Pak	UHT Milk	200 mL	3.5%
Sample 51	Tetra Pak	UHT Milk	200 mL	1.5%

Table S2. Some information about the beverage samples

Sample No	Packaging type	Product features	Consumption amount	Fat content according to label information
Sample 52	Plastic	Soft drink (blue)	330 mL	0
Sample 53	Plastic	Soft drink (cola)	330 mL	0
Sample 54	Plastic	Soft drink (flavored soda)	330 mL	0
Sample 55	Plastic	Soft drink (orange-flavored)	330 mL	0
Sample 56	Plastic	Soft drink (orange-flavored)	330 mL	0
Sample 57	Plastic	Soft drink (orange-flavored)	330 mL	0
Sample 58	Plastic	Soft drink (orange-flavored)	330 mL	0
Sample 59	Plastic	Soft drink (cola)	330 mL	0
Sample 60	Plastic	Soft drink (flavored soda)	330 mL	0
Sample 61	Plastic	Soft drink (cola)	330 mL	0
Sample 62	Plastic	Soft drink (cola)	330 mL	0
Sample 63	Tin	Soft drink (cola)	330 mL	0
Sample 64	Tin	Soft drink (orange-flavored)	330 mL	0
Sample 65	Tin	Soft drink (cola)	330 mL	0
Sample 66	Tin	Soft drink (cola)	330 mL	0
Sample 67	Tin	Soft drink (orange-flavored)	330 mL	0
Sample 68	Tin	Soft drink (flavored soda)	330 mL	0
Sample 69	Tin	Soft drink (cola)	330 mL	0
Sample 70	Tin	Soft drink (cola)	330 mL	0
Sample 71	Tin	Soft drink (flavored soda)	330 mL	0
Sample 72	Tin	Soft drink (flavored soda)	330 mL	0
Sample 73	Tin	Soft drink (flavored soda)	330 mL	0
Sample 74	Tin	Soft drink (cola)	330 mL	0
Sample 75	Tin	Soft drink (orange-flavored)	330 mL	0
Sample 76	Plastic	Fruit juice (cherry)	200 mL	0
Sample 77	Plastic	Fruit juice (orange)	200 mL	0
Sample 78	Tetra Pak	Fruit juice (mixed)	200 mL	0
Sample 79	Tetra Pak	Fruit juice (mixed)	200 mL	0.2%
Sample 80	Tetra Pak	Fruit juice (mixed)	200 mL	0.1%
Sample 81	Tetra Pak	Fruit juice (cherry)	200 mL	0.2%
Sample 82	Tetra Pak	Fruit juice (cherry)	200 mL	0
Sample 83	Tetra Pak	Fruit juice (apricot)	200 mL	0
Sample 84	Tetra Pak	Fruit juice (mixed)	200 mL	0.1%
Sample 85	Tetra Pak	Fruit juice (mixed)	200 mL	0.2%
Sample 86	Tetra Pak	Fruit juice (peach)	200 mL	0
Sample 87	Tetra Pak	Fruit juice (cherry)	200 mL	0
Sample 88	Tetra Pak	Fruit juice (apricot)	200 mL	0.1%
Sample 89	Tetra Pak	Fruit juice (apricot)	200 mL	0
Sample 90	Tetra Pak	Fruit juice (orange)	200 mL	0
Sample 91	Plastic	Energy drink	330 mL	0
Sample 92	Tin	Energy drink	330 mL	0
Sample 93	Tin	Energy drink	330 mL	0
Sample 94	Tin	Energy drink	330 mL	0
Sample 95	Tin	Energy drink	330 mL	0
Sample 96	Tin	Energy drink	330 mL	0
Sample 97	Tin	Energy drink	330 mL	0
Sample 98	Tin	Energy drink (vitamin C)	330 mL	0
Sample 99	Tin	Energy drink (mutlivitamin)	330 mL	0
Sample 100	Plastic	Bottled water	200 mL	0
Sample 101	Plastic	Bottled water	200 mL	0
Sample 102	Plastic	Bottled water	200 mL	0
Sample 103	Plastic	Bottled water	200 mL	0
Sample 104	Plastic	Bottled water	200 mL	0
Sample 105	Plastic	Bottled water	200 mL	0
Sample 106	Plastic	Bottled water	200 mL	0
Sample 107	Plastic	Bottled water	200 mL	0
Sample 108	Tin	Cold coffee (latte)	250 mL	0.9%
Sample 109	Tin	Cold coffee (latte)	250 mL	1.1%
Sample 110	Tin	Cold coffee (latte)	250 mL	0.9%
Sample 111	Tin	Cold coffee (latte)	250 mL	1.1%
Sample 112	Tin	Cold coffee (cappucino)	250 mL	1.9%
Sample 113	Tin	Cold coffee (cappucino)	250 mL	1.1%
Sample 114	Tin	Cold coffee (mocca)	250 mL	1.8%
Sample 115	Tin	Cold coffee (latte)	250 mL	0.9%
Sample 116	Tin	Ice tea	330 mL	0
Sample 117	Tin	Ice tea	330 mL	0
Sample 118	Tin	Ice tea	330 mL	0
Sample 119	Tin	Ice tea	330 mL	0

Sample 120	Tin	Ice tea	330 mL	0
Sample 121	Tin	Ice tea	330 mL	0
Sample 122	Tin	Ice tea	330 mL	0
Sample 123	Plastic	Lemonade	200 mL	0
Sample 124	Plastic	Lemonade	200 mL	0
Sample 125	Plastic	Lemonade	200 mL	0
Sample 126	Plastic	Lemonade	200 mL	0
Sample 127	Plastic	Traditional turnip juice	200 mL	0.1%
Sample 128	Plastic	Traditional turnip juice	200 mL	0.1%
Sample 129	Plastic	Traditional turnip juice	200 mL	0
Sample 130	Plastic	Traditional turnip juice	200 mL	0

Table S3. GC analysis program for PCBs

**Device Model**	Thermo GC/MS-MS TSQ 8000 Triple Quadrupole MS		
	**Rate**	**Temperature (°C)**	**Time (min.)**
**Injection**		60	0.1
**Transfer**	8	280	4
**Cleaning**	14.5	330	35
**Carrier Gas**	Helium		
**Carrier Gas Flow**	1,3 mL/min.		
**Column Type**	Thermo TraceGOLD™ TG-5 SILMS		
**Column Length**	30 m		
**Column inn. Diameter**	0.25 mm		
**Film Thickness**	0.25 µm		
**Detector Type**	MS-MS		
**Detector Temperature**	280°C		
**Electron Energy**	70 ev		
**Injection Volume**	2 µL		
**Detector Gain Factor**	7		
**Oven Temperature Program**	**Rate**	**Temperature (°C)**	**Time (min.)**
**Start**		80	2
	30	150	0.01
	5	290	0
	30	310	1
**Inlet**	**Split Rate**	**Temperature (°C)**	**Split Flow (mL/min.)**
	7,07	60	10
**Surge Pressure**	250 kPa		
**Surge Time**	2 min.		
**Septum Purge Flow**	5 mL/min.		

Table S4. GC-MSMS method parameters for each PCB analytes

Analytes	RT (min)	Ion Polarity	Target ion (m/z)	Precursor ion 1 (m/z)	Precursor ion 2 (m/z)	Collision energy (eV)
**PCBs**						
PCB 29*	13.70	Positive	256	151	186	40
PCB 28	14.24	Positive	256	186	186	20
PCB 52	15.44	Positive	290	220	220	20
PCB 101	18.47	Positive	324	254	256	20
PCB 118	20.69	Positive	324	254	256	20
PCB 153	21.46	Positive	358	288	290	25
PCB 138	22.38	Positive	358	288	290	25
PCB 180	24.75	Positive	392	322	324	25
PCB 198*	25.87	Positive	498	360	395	25

* IS

Table S5. PCBs levels in milk and dairy products (mean±SD; min−max) (μg/L wet weight)

Type of dairy products	PCB 28	PCB 52	PCB 101	PCB 118	PCB 138	PCB 153	PCB 180	ICES-7
UHT Milk	0.01±0.01 (N.D.−0.01)	0.01±0.01 (N.D.−0.02)	0.01±0.01 (N.D.−0.04)	<LOQ (N.D.−0.02)	0.01±0.01 (N.D.−0.05)	0.02±0.03 (N.D.−0.09)	0.02±0.04 (N.D.−0.13)	0.07±0.10 (N.D.−0.36)
Children's milk	0.01±0.01 (N.D.−0.01)	0.01±0.01 (N.D.−0.03)	0.01±0.02 (N.D.−0.05)	<LOQ (N.D.−0.01)	0.01±0.01 (N.D.−0.04)	0.01±0.02 (N.D.−0.07)	0.02±0.03 (N.D.−0.11)	0.05±0.08 (N.D.−0.32)
Ayran	<LOQ (N.D.−0.01)	<LOQ (N.D.−0.01)	<LOQ (N.D.−0.01)	<LOQ N.D.	<LOQ (N.D.−0.01)	0.01±0.01 (N.D.−0.02)	0.01±0.01 (N.D.−0.01)	0.02±0.02 (N.D.−0.07)
Yogurt	0.01±0.01 (N.D.−0.01)	0.01±0.01 (N.D.−0.01)	0.01±0.01 (N.D.−0.01)	0.01±0.01 (N.D.−0.05)	0.01±0.01 (N.D.−0.03)	0.01±0.02 (N.D.−0.06)	0.01±0.02 (N.D.−0.06)	0.06±0.07 (N.D.−0.23)
Kefir	0.01±0.01 (N.D.−0.01)	0.01±0.01 (N.D.−0.01)	0.01±0.01 (N.D.−0.02)	0.01±0.01 (N.D.−0.02)	0.01±0.01 (N.D.−0.03)	0.02±0.03 (N.D.−0.07)	0.01±0.02 (N.D.−0.06)	0.07±0.08 (N.D.−0.22)

N.D.: Not detected.

Table S6. PCBs levels in beverages (mean±SD; min−max) (µg/L wet weight)

Type of beverages	PCB 28	PCB 52	PCB 101	PCB 118	PCB 138	PCB 153	PCB 180	ICES-7
Soft drink	<LOQ (N.D.−0.01)	0.01±0.01 (N.D.−0.02)	0.01±0.01 (N.D.−0.03)	N.D.	<LOQ (N.D.−0.02)	0.01±0.01 (N.D.−0.04)	0.01±0.01 (N.D.−0.05)	0.04±0.05 (N.D.−0.17)
Lemonade	<LOQ (N.D.−<LOQ)	<LOQ (N.D.−<LOQ)	<LOQ (N.D.−<LOQ)	N.D.	N.D.	<LOQ (N.D.−<LOQ)	<LOQ (N.D.−<LOQ)	<LOQ (N.D.−<0.02)
Ice tea	<LOQ (N.D.−0.01)	<LOQ (N.D.−0.01)	0.01±0.01 (N.D.−0.02)	<LOQ (N.D.−0.01)	0.01±0.01 (N.D.−0.02)	0.01±0.01 (N.D.−0.03)	0.01±0.01 (N.D.−0.04)	0.03±0.04 (N.D.−0.14)
Energy drink	<LOQ (N.D.−0.01)	<LOQ (N.D.−<LOQ)	<LOQ (N.D.−<LOQ)	N.D.	<LOQ (N.D.−<LOQ)	<LOQ (N.D.−0.01)	<LOQ (N.D.−0.01)	0.01±0.02 (N.D.−0.03)
Fruit juice	<LOQ (N.D.−0.01)	<LOQ (N.D.−0.02)	0.01±0.01 (N.D.−0.05)	N.D.	<LOQ (N.D.−0.03)	0.01±0.02 (N.D.−0.08)	0.01±0.02 (N.D.−0.08)	0.03±0.06 (N.D.−0.27)
Traditional turnip juice	0.01±0.01 (N.D.−0.02)	0.01±0.01 (N.D.−0.03)	0.01±0.02 (N.D.−0.04)	N.D.	0.01±0.01 (N.D.−0.02)	0.01±0.02 (N.D.−0.03)	0.01±0.02 (N.D.−0.03)	0.04±0.08 (N.D.−0.17)
Cold coffee	0.01±0.01 (N.D.−0.02)	0.01±0.01 (N.D.−0.02)	0.01±0.01 (N.D.−0.02)	0.01±0.02 (N.D.−0.05)	0.01±0.01 (N.D.−0.03)	0.01±0.02 (N.D.−0.06)	0.01±0.02 (N.D.−0.05)	0.05±0.08 (N.D.−0.25)
Bottled water	<LOQ N.D.−0.01)	0.01±0.01 (N.D.−0.02)	0.01±0.01 (N.D.−0.04)	<LOQ (N.D.−0.02)	0.01±0.02 (N.D.−0.06)	0.02±0.02 (N.D.−0.06)	0.01±0.02 (N.D.−0.06)	0.06±0.08 (N.D.−0.27)

N.D.: Not detected.

Table S7. PCBs levels in packaging types (mean±SD; min−max) (µg/L wet weight)

Products	Type of packaging	PCB 28	PCB 52	PCB 101	PCB 118	PCB 138	PCB 153	PCB 180	ICES-7
Beverages	Tin	<LOQ (N.D.−0.02)	0.01±0.01 (N.D.−0.02)	0.01±0.01 (N.D.−0.03)	<LOQ (N.D.−0.05)	<LOQ (N.D.−0.03)	0.01±0.01 (N.D.−0.06)	0.01±0.01 (N.D.−0.05)	0.04±0.05 (N.D.−0.26)
	Plastic	<LOQ (N.D.−0.02)	0.01±0.01 (N.D.−0.03)	0.01±0.01 (N.D.−0.04)	<LOQ (N.D.−0.02)	0.01±0.01 (N.D.−0.06)	0.01±0.01 (N.D.−0.06)	0.01±0.01 (N.D.−0.06)	0.05±0.06 (N.D.−0.29)
	Tetra Pak	<LOQ (N.D.−0.01)	<LOQ (N.D.−0.02)	0.01±0.01 (N.D.−0.05)	N.D.	<LOQ (N.D.−0.03)	0.01±0.02 (N.D.−0.07)	0.01±0.02 (N.D.−0.08)	0.03±0.06 (N.D.−0.26)
Milk and dairy products	Plastic	0.01±0.01 (N.D.−0.01)	0.01±0.01 (N.D.−0.01)	0.01±0.01 (N.D.−0.02)	0.01±0.01 (N.D.−0.05)	0.01±0.01 (N.D.−0.03)	0.01±0.02 (N.D.−0.07)	0.01±0.02 (N.D.−0.06)	0.06±0.07 (N.D.−0.25)
	Tetra Pak	0.01±0.01 (N.D.−0.02)	0.01±0.01 (N.D.−0.03)	0.01±0.01 (N.D.−0.05)	0.01±0.01 (N.D.−0.05)	0.01±0.02 (N.D.−0.05)	0.02±0.03 (N.D.−0.09)	0.02±0.03 (N.D.−0.13)	0.07±0.10 (N.D.−0.42)

N.D.: Not detected.
